# Association between metabolic syndrome and bone fractures: a meta-analysis of observational studies

**DOI:** 10.1186/1472-6823-14-13

**Published:** 2014-02-09

**Authors:** Kan Sun, Jianmin Liu, Nan Lu, Hanxiao Sun, Guang Ning

**Affiliations:** 1 Department of Endocrinology and Metabolism, Rui-Jin Hospital, Shanghai Jiao Tong University School of Medicine, Shanghai Institute of Endocrine and Metabolic Diseases, Shanghai Clinical Center for Endocrine and Metabolic Diseases, Shanghai 200025, China; 2 Key Laboratory for Endocrine and Metabolic Diseases of Ministry of Health, Rui-Jin Hospital, Shanghai Jiao Tong University School of Medicine, E-Institute of Shanghai Universities, Shanghai 200025, China

**Keywords:** Metabolic syndrome, Fractures, Cohort study, Cross-sectional study, Meta-analysis

## Abstract

**Background:**

Emerging epidemiological evidence suggest an association between metabolic syndrome and fractures. However, whether metabolic syndrome is an independent risk or protective factor of fractures remains controversial. Our goal is to provide a quantitative assessment of the association between metabolic syndrome and bone fractures by conducting a meta-analysis of observational studies.

**Methods:**

The PubMed and Embase database were searched through to March 2013 to identify studies that met pre-established inclusion criteria. Reference lists of retrieved articles were also reviewed. Summary effect estimates with 95% confidence intervals (CI) were derived using a fixed or random effects model, depending on the heterogeneity of the included studies.

**Results:**

Eight epidemiologic studies involving 39,938 participants were included in the meta-analysis. In overall analysis, metabolic syndrome was not associated with prevalent fractures [pooled odds ratio (OR) 0.93, 95% CI 0.84 - 1.03] in cross-sectional studies or incident fractures [pooled relative risk (RR) 0.88, 95% CI 0.37 - 2.12] in prospective cohort studies. No evidence of heterogeneity was found in cross-sectional studies (*p* = 0.786, *I*^*2*^ = 0.0%). A substantial heterogeneity was detected in cohort studies (*p* = 0.001, *I*^*2*^ = 85.7%). No indication of significant publication bias was found either from Begg’s test or Egger’s test. Estimates of total effects were substantially consistent in the sensitivity and stratification analyses.

**Conclusions:**

The present meta-analysis of observational studies suggests that the metabolic syndrome has no explicit effect on bone fractures.

## Background

Metabolic syndrome includes the constellation of various metabolic abnormalities and confers an increased risk for diabetes and cardiovascular diseases. It is becoming a global burden because the prevalence of metabolic syndrome in adult was around 20-25% all over the world [1]. Osteoporotic fractures among the older people are also a major health problem leading to increased mortality and morbidity and significant costs on public-health budgets [2,3].

The association between metabolic syndrome and osteoporotic fractures has been analyzed in recent epidemiological studies, but the results are quite discordant. Muhlen D et al. [4] found that incidence of osteoporotic non-vertebral fractures was higher in participants with metabolic syndrome in the Rancho Bernardo Study. However, as reported in the Third National Health and Nutrition Examination Survey (NHANES III), no difference in prevalence of non-vertebral fractures was found between people with and without metabolic syndrome [5]. Similarly on non-vertebral fractures, Luai A. Ahmed et al. [6] found that metabolic syndrome have a significant protective effect on its risk in the Tromsø Study. Recently, Pawel Szulc et al. [7] found that men with metabolic syndrome have lower bone mineral density (BMD) but lower fracture risk in the MINOS study. Nevertheless, a later meta-analysis suggests that metabolic syndrome has no clear influence on BMD, or its influence maybe beneficial [8].

Combined with early epidemiological studies, the real pattern of metabolic syndrome on fractures has not been clearly elucidated. Moreover, previously existing primary analyses on this association did not complete a meta-analysis of data sources. Therefore, the present meta-analysis of the literature aims to obtain an overview of metabolic syndrome as a concept in the context of fractures.

## Methods

### Search strategy

We conducted a systematic review of the published works without language restrictions and in accordance with the Meta-analysis of Observational Studies in Epidemiology (MOOSE) guidelines [9]. We searched Pubmed and Embase from their inception to March 2013 and systematically identified observational studies that evaluated the association between metabolic syndrome and incidence or prevalence of fractures. We used the following main search terms with no restrictions: “metabolic syndrome” or “insulin resistance syndrome” or “syndrome X” in combination with “bone” or “fracture” or “osteoporosis” or “metabolic bone diseases ” or “osteopenia” or “bone mineral density” or “BMD”. We also scanned the reference lists from published original articles and previous reviews for more relevant studies not identified in the databases search.

### Study selection

We included studies in the meta-analysis that met all of the following criteria: (1) the study had a population based observational design, (2) published original data relevant to a possible association between metabolic syndrome and fractures, (3) reported the odds ratio (OR) or relative risk (RR) and its 95% confidence interval (CI). In case of multiple publications had overlap their populations and reported with the same study design, the most recent publication was included in order to avoid duplicate observation, unless more inclusive and detailed data was found in other publications. To gather more relevant information, we consulted researchers with professional knowledge at this area for the presence of unpublished reports.

### Data extraction

Two of our reviewers independently evaluated all relevant articles and identified eligible studies from the databases. During data abstraction, differences and disagreements were resolved through discussion to come to an agreement. Following information was recorded by a standardized data extraction form: last name of the first author, publication year, name of the study, geographic region of original study, composition and age range of study population, type of fractures, definitions of metabolic syndrome, unadjusted and adjusted OR or RR with corresponding 95% CI and adjustment factors of interest. If possible, we also extracted the baseline data of cohort studies for the combined estimates. We contacted authors of the primary studies for additional information when necessary.

### Statistical analysis

The primary outcome of the pooled analysis was focused on a comparison of the summary effect of fractures risk in people with metabolic syndrome versus those without. Both unadjusted and adjusted values were extracted for the pooled analysis. When studies presented results from various covariates analyses, we used the one adjusted the most study-specific confounders. The combined estimates were calculated separately by averaging the natural logarithmic OR or RR weighted by their inverse of variance based on a fixed or random effects model within or between study variations, depending on the overall heterogeneity. Heterogeneity of effect size across studies was assessed by using Cochran’s Q and the *I*^*2*^ statistic [10,11] and *p* value < 0.10 or *I*^*2*^ value > 50% was considered to be heterogeneous. In the *I*^*2*^ statistic, values of 25, 50, and 75% are considered to represent low, medium, and high heterogeneity, respectively. If substantial heterogeneity was detected, pooled effect estimates were calculated using a random effects model by the method of DerSimonian and Laird [12]. If not, the combined estimates were presented based on the fixed effects model by using the inverse variance method [13].

To assess the influence of individual studies on the pooled result, we conducted sensitivity analyses to investigate the influence of a single study on the overall risk estimate by omitting one study in each turn. We used the Begg’ s adjusted rank correlation test and the Egger’ s regression asymmetry test to detect publication bias and *p* > 0.05 for both tests was considered to be no significant publication bias [14,15]. Subgroup analyses according to sex (male/female), types of fractures (non-vertebral/vertebral/any fractures), definitions of metabolic syndrome [strict National Cholesterol Education Program’ s Adult Treatment Panel III (NCEP-ATP III) criteria-2001/strict NCEP-ATP III criteria-2005/other modified criteria] and geographical area (Europe/America/Asia) were used to assess the impacts of study characteristics on outcomes.

All statistical analyses were performed using STATA version 11.0 (Stata Corp, College Station, TX, USA).

## Results

**Figure 1:**
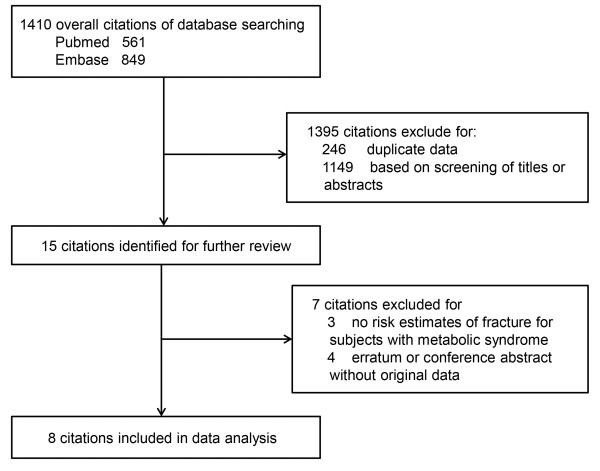
Flow diagram of included studies in the systematic review.

The details of the literature search were presented in a flow diagram (Figure 1). We identified 1,410 citations (561 from Pubmed and 849 from Embase) with the electronic literature search. We first excluded 246 citations for the duplicate data in the databases. Then we excluded 1,149 citations by screening abstracts or titles. After this, fifteen remaining citations were identified for further full-text reviewed. Finally, eight citations met the inclusion criteria were included in the data analysis.

The characteristics and information of the included studies were showed in Table 1. Totally, the 8 selected studies contained 39,938 participants. Among the 8 included studies, five studies showed no significant correlation between metabolic syndrome and prevalent fractures [5,16-19]. However, in the Rancho Bernardo Study, incidence of osteoporotic fractures was significant higher in patients with metabolic syndrome compared to those without [4]. By contrast, in the Tromsø prospective study, a significant protective effect of metabolic syndrome against non-vertebral fractures was found in both sexes [6]. Metabolic syndrome was associated with a lower fracture incidence in the MINOS study [7].

**Table 1:** Characteristics of included studies

**Source**	**Region**	**Study name**	**Study type**^**&**^	**Paticipants (new cases)**	**Age range (years)**	**Metabolic syndrome measures**	**Type of fractures**
Ahmed [6]	Norway	The Tromsø Study	Prospective	All: 26,905*	25 - 98	NCEP-ATP III	Non-vertebral fractures
			(6 years)	(1,227)			
Mitsuyo [5]	USA	NHANES III	Cross-sectional	Male: 4,026	20 or older	NCEP-ATP III	Non-vertebral fractures
				Female: 4,171			
von Muhlen [4]	USA	The Rancho Bernardo Study	Prospective (2 years)	Male: 420 (9)	Male: 74.2 ± 9.7	NCEP-ATP III	Non-vertebral fractures
				Female: 676 (22)	Female: 74.4 ± 10.9		
Yamaguchi [16]	Japan	N.A.^#^	Cross-sectional	Male: 187	Male: 59.7 ± 13.5	Japanese criteria	Vertebral fractures
				Female: 125	Female: 64.7 ± 10.9		
Hernández [17]	Spain	The Camargo Cohort Study	Cross-sectional	Male: 495	Male: 65.0 ± 9.0	NCEP-ATP III	Any fractures
				Female: 1,013	Female: 63.0 ± 9.0		
Kim [18]	South Korea	N.A.	Cross-sectional	Female: 907	60 - 79	NCEP-ATP III	Vertebral fractures
Szulc [7]	France	The MINOS Study	Prospective (10 years)	Male: 762 (82)	50 - 85	NCEP-ATP III	Any fractures
Ferre [19]	Spain	The PREDIMED Study	Cross-sectional	Male: 124	Male: 55–80	NCEP-ATP III	Any fractures
				Female: 127	Female: 60 - 80		

^&^Follow-up duration (years) if a prospective design.

*Relevant data from erratum on Osteoporos Int [6] 20:839.

^#^N.A., not recorded or available.

**Figure 2:**
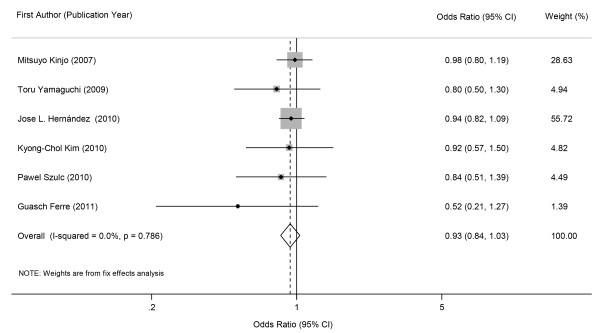
Forest plot showing combined estimates of metabolic syndrome and prevalence of fractures.

**Figure 3:**
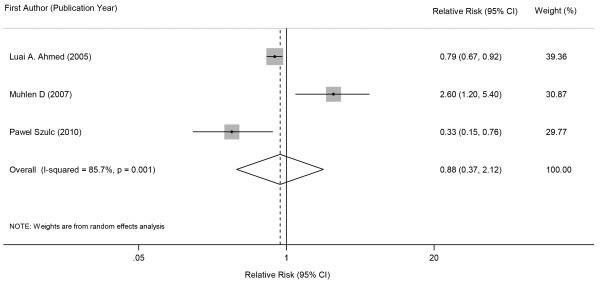
Forest plot showing combined estimates of metabolic syndrome and incidence of fractures.

In overall analysis of the 8 selected studies, metabolic syndrome was not associated with prevalent fractures in cross-sectional studies (Figure 2, pooled OR 0.93, 95% CI 0.84 - 1.03) or incident fractures in prospective cohort studies (Figure 3, pooled RR 0.88, 95% CI 0.37 - 2.12). Statistically significant evidence of heterogeneity was found in cohort studies (*p* = 0.001, *I*^*2*^ = 85.7%) but not in cross-sectional studies (*p* = 0.786, *I*^*2*^ = 0.0%). There was no indication of significant publication bias either from the result of Begg’s test (*p* = 0.133 for cross-sectional studies and *p* = 1.000 for prospective cohort studies) or from the Egger’s test (*p* = 0.054 for cross-sectional studies and *p* = 0.893 for prospective cohort studies).

Sensitivity analyses of our primary outcome were carried out by excluding one study at a time and the combined overall risk estimates were consistent, with a range from 0.91 (95% CI 0.80 - 1.03) to 0.94 (95% CI 0.84 - 1.05) in cross-sectional studies and 0.56 (95% CI 0.24 - 1.29) to 1.35 (95% CI 0.42 - 4.32) in prospective cohort studies, respectively. As only 3 prospective cohort studies were included in the meta-analysis, we conducted the subgroup analyses by only adopting the cross-sectional data. As shown in Table 2, the results of subgroup analyses according to different characteristics are in close agreement with our major finding.

**Table 2:** Stratified risk estimates of the association between metabolic syndrome and bone fractures

**Group**	**NO. of studies**	**OR (95% CI)**	***p ***_**for heterogeneity**_	***I***^**2 **^**(%)**
Sex				
Male	3	0.89 (0.72 - 1.11)	0.254	27
Female	3	0.94 (0.81 - 1.10)	0.614	0.0
Type of fractures				
Non-vertebral fractures	1	0.98 (0.80 - 1.20)	--	--
Vertebral fractures	2	0.86 (0.61 - 1.20)	0.687	0.0
Any fractures*	3	0.92 (0.80 - 1.05)	0.415	0.0
Definitions of metabolic syndrome				
NCEP-ATP III criteria-2001	3	0.96 (0.80 - 1.13)	0.844	0.0
NCEP-ATP III criteria-2005	2	0.93 (0.81 - 1.07)	0.203	38.4
Other criteria	1	0.80 (0.50 - 1.29)	--	--
Geographical area				
Europe	3	0.92 (0.80 - 1.05)	0.415	0.0
America	1	0.98 (0.80 - 1.20)	--	--
Asia	2	0.86 (0.61 - 1.20)	0.687	0.0

*Specific classification of fractures was not available in these studies.

## Discussion

According to the observational data in the past decade, the relationship between metabolic syndrome and osteoporotic fractures is controversial. Findings of the present meta-analysis show no clear link between metabolic syndrome and fractures. The combined estimate of our primary analysis is strong across sensitivity analyses and without significant publication bias. Despite the limited numbers of studies in this area, we found that cross-sectional studies yield estimates of the pooled effect of metabolic syndrome on bone fractures were in agreement with those from prospective cohort studies.

The influence of metabolic syndrome on bone fractures is complicated. When explored such association, we should notice that the two diseases states share similar risk factors, such as aging, physical inactivity, cigarette smoking and alcohol drinking [20-23]. The occurrence of fractures may be directly mediated by exposure of these risk factors, not by metabolic syndrome itself. Moreover, the diagnosis of metabolic syndrome is the inclusion of various combinations as a whole, which result in subjects with the disease becoming a considerably heterogeneous group. Therefore, when addressing the relationship between metabolic syndrome and fractures, it should be more appropriate to evaluate the effects of individual components of metabolic syndrome on bone fractures.

Previous studies have shown that elevated blood pressure is a risk factor for fractures [24]. However, as another component of the metabolic syndrome, hypertriglyceridemia contribute to a lower risk of low-trauma fractures, which may partly because triglycerides mediate the interaction between the protein matrix and bone minerals [7,25]. Moreover, lower serum levels of high density lipoprotein may protect against fractures in women and obese men [6]. Some of other metabolic syndrome components may just play a discordant role in the relationship between metabolic syndrome and fractures. Metabolic syndrome is a risk factor for diabetes which might expect an increase in fracture risk. Actually, patients with type 2 diabetes had an increased fracture risk in spite of a higher BMD level, which may partly cause by the increased risk of falling [26]. Strotmeyer et al. [27] found that subjects with type 2 diabetes mellitus may suffer from higher risk of osteoporotic fractures. However, impaired fasting plasma glucose is not associated with fracture risk in the same study. Gagnon C et al. [28] reported that incident fractures were reduced in individuals with elevated 2 hours plasma glucose levels and pre-diabetes independently of BMI and fasting insulin levels. Therefore, increased awareness of the association between metabolic syndrome and fractures is still needed in view of the above argument and growing population of patients with diabetes and impaired glucose regulation [29]. In addition, obesity is a risk factor for fractures of the humerus and ankle but protects against fractures of hip and vertebral [30-32]. Additionally, the association between obesity and fractures appears to vary with age. Dimitri et al. [33] found that obesity is a risk factor of fractures in children but a protective factor of fractures in adults. Future studies are needed to search for more credible evidence and identify the exact mechanisms that link obesity to fracture risk [34]. Base on the above arguments, we assume that the concept of metabolic syndrome may be slightly far-fetched in the context of fractures. The controversial results of the included studies in this meta-analysis may mainly depend on the discrepancy between each individual component of metabolic syndrome and bone fractures. Moreover, the negative findings of current meta-analysis, to some extent, just verify this viewpoint.

There were several limitations to this meta-analysis. First, the distinct definition of metabolic syndrome might provide biased estimates on bone fractures. Moreover, the Tromsø Study took non-fasting samples for testing glucose and triglycerides levels and used BMI instead of waist circumference to define metabolic syndrome. Actually, a variety of definitions of metabolic syndrome has existed for at least ten decades. Among these definitions, the NCEP-ATP III definition is widely used in both clinical and epidemiological studies. In the current analysis, nevertheless, metabolic syndrome was defined by rigorous NCEP-ATP III criteria in most of the included studies and pooled results of prevalent fractures were kept consistent in subgroup analyses. Second, the present analysis may be subject to recall bias of fractures because most studies included participants 50 years or older. However, fracture is one of the major health events in life and rarely to be forgotten or misremembered. Second, the availability of articles in this area was relatively limited. To better elucidate the association between metabolic syndrome and fractures, we included both cross-sectional and prospective cohort studies in the present meta-analysis. The inclusion of cross-sectional studies may result in high potential for intractable confounding and reverse causation. However, the pooled results from cross-sectional studies were in close agreement with those from prospective cohort studies and no evidence of heterogeneity was found in cross-sectional studies. Third, although most of the included studies made attempt to control for the confounding variables, not all of the residual and potential mediators were adjusted and took into account, which could contribute to a superficially conclusion of our findings. Finally, a substantial degree of heterogeneity, partly due to diversity in fractures types and the duration of follow up, was detected among cohort studies. For example, fractures in the Tromsø Study were not limited to non-trauma fractures. Moreover, the Rancho Bernardo Study found an increased risk of fractures in people with metabolic syndrome had only 2 years of follow-up. In the Tromsø Study and the MINOS Study, had a longer follow-up of 6 and 10 years, respectively, found a reduced risk of fractures in people with metabolic syndrome. However, such heterogeneity was not surprising because of unavoidable variations in study population and distinct adjustments across studies. Moreover, overall risk estimates of cohort studies did not substantially modified through the sensitivity analyses.

## Conclusion

In conclusion, the present meta-analysis collected and synthesized data currently available and found that metabolic syndrome is not clearly associated with prevalence or incidence of fractures. In consideration of the high heterogeneity of the prospective studies and limitations of the data consolidation, further longitudinal studies are urgently needed to make definite conclusion on this issue.

## Competing interests

All of the authors have no relevant conflict of interests.

## Authors’ contributions

KS writes the paper, KS and JL conceived and carried out data analysis. NL verified all data analysis. HS and GN modified and reviewed the article. All authors had final approval of the submitted and published versions.

## Pre-publication history

The pre-publication history for this paper can be accessed here:

http://www.biomedcentral.com/1472-6823/14/13/prepub
